# The right temporoparietal junction and cooperation dilemma

**DOI:** 10.1192/j.eurpsy.2021.1952

**Published:** 2021-08-13

**Authors:** S. Tei, J. Fujino

**Affiliations:** 1 Department Of Psychiatry, Kyoto University, Kyoto, Japan; 2 Institute Of Applied Brain Sciences, Waseda University, Saitama, Japan; 3 School Of Human And Social Sciences, Tokyo International University, Saitama, Japan; 4 Medical Institute Of Developmental Disabilities Research, Showa University, Tokyo, Japan; 5 Department Of Psychiatry And Behavioral Sciences, Tokyo Medical and Dental University, Tokyo, Japan

**Keywords:** temporoparietal junction, cooperation, transcranial magnetic stimulation, decision making

## Abstract

**Introduction:**

Cooperation is a key component of our lives. When we identify people in need, we are frequently motivated to cooperate by overcoming selfishness. However, we may also become selfish to pursue greater gains by putting ourselves at risk and exploiting others. Such cooperation dilemmas are ubiquitous in real life. Although functional magnetic resonance imaging studies have repeatedly reported the involvement of right temporoparietal junction (rTPJ) in cooperation dilemmas, a causal link between the two has been rarely explored.

**Objectives:**

To investigate a causal role of rTPJ in resolving cooperation dilemmas in ecologically valid settings.

**Methods:**

Twenty-two healthy volunteers were examined. We combined repetitive transcranial magnetic stimulation (rTMS) with a snowdrift cooperation dilemma game task (cross-the-traffic intersection version) wherein either cooperation or defection should be chosen. Participants and opponents jointly faced a problem at the intersection where their cooperation could diffuse the situation (stopping/avoiding a car-crash). This conflicted with a choice in the participant’s self-interest which was more rewarding, but risky (not stopping/defection). We also included explicit-cue condition that showed elderly/pregnant passengers in the opponent’s car. Furthermore, we measured participants’ empathic-traits (e.g., perspective-taking) to study personality-cooperation associations.

**Results:**

The cooperation-ratio did not statistically differ between the sham stimulation and inhibitory continuous theta burst stimulation (cTBS) in both the no-cue and with-cue conditions. However, after cTBS, only in the no-cue condition, the strength of the relationship between cooperation-ratios and empathic-traits decreased significantly (p<0.05).

**Conclusions:**

These results contribute to our understandings of rTPJ’s role in spontaneous social cognition, which may be considerably complex and require further examination.

**Disclosure:**

No significant relationships.

